# Tuberculosis in times of COVID-19: we cannot lose focus on the diagnosis

**DOI:** 10.1590/0100-3984.2022.55.2e1

**Published:** 2022

**Authors:** Pedro Paulo Teixeira e Silva Torres, Marcelo Fouad Rabahi

**Affiliations:** 1 Doutorando em Radiologia na Universidade Federal do Rio de Janeiro (UFRJ), Rio de Janeiro, RJ, Médico Radiologista Torácico do Hospital Israelita Albert Einstein e da Multimagem Diagnósticos, Goiânia, GO, Brasil; 2 Universidade Federal de Goiás e Hospital Israelita Albert Einstein, Goiânia, GO, Brasil.

In patients with coronavirus disease 2019 (COVID-19), especially in those with the severe form of the disease, coinfection (including infection with bacteria, fungi, and even other viruses) is a common occurrence, bacterial coinfection reportedly occurring in up to 14.3% of critically ill patients with COVID-19^(1)^. In the previous issue of Radiologia Brasileira, an article authored by Mançano et al.^(2)^ addresses the exceptional relationship between two distinct, serious, urgent public health problems, in Brazil and in the world: one that has been around for millennia (tuberculosis); and one that has arisen recently (COVID-19). It is estimated that approximately 2 billion people (one quarter of the world population) are currently infected with tuberculosis, and that there have been approximately 282 million confirmed cases of COVID-19 to date^(3,4)^. A cursory comparison between the two diseases allows us to perceive similarities, such as airborne transmission, analogous symptoms (cough and fever), the potential for structural lung sequelae, and a social stigma. However, there are also marked differences, such as the fact that COVID-19 usually has a benign course in children, whereas, in 2020, 16% of all tuberculosis deaths among HIV-negative individuals occurred in children^(3,5)^.

From the perspective of tuberculosis as a serious global public health problem, the immediate consequence of the COVID-19 pandemic was a reduction in the number of new cases reported. Although the number of reported cases of tuberculosis worldwide increased between 2017 and 2019, there was an 18% reduction (from 7.1 million to 5.8 million) between 2019 and 2020, Brazil being among the countries that made the greatest contribution to creating that scenario^(3)^. Concomitantly, there was an increase in the global, regional, and national rates of tuberculosis-related mortality, reversing years of progress in reducing the number of deaths from the disease^(3)^.

At the level of the individual, the most relevant aspect is the possibility of greater severity and mortality in patients coinfected with severe acute respiratory syndrome coronavirus 2 (SARS-CoV-2) and *Mycobacterium tuberculosis*^(1,6,7)^. The meta-analyses and systematic reviews conducted by Sarkar et al.^(1)^ and Aggarwal et al.^(7)^ showed an approximately two-fold greater risk of mortality in patients coinfected with *M. tuberculosis* and SARS-CoV-2 than in those infected with SARS-CoV-2 alone. That increased risk is similar to that reported for other comorbidities known to negatively affect the prognosis of patients with COVID-19, such as diabetes, hypertension, and cardiovascular disease^(7)^. However, in another systematic review and meta-analysis, Gao et al.^(6)^ found no statistically significant difference in mortality between COVID-19 patients who were coinfected with *M. tuberculosis* and those who were not. The situations that could worsen the outcome of such coinfection include advanced age, male gender, advanced manifestations of tuberculosis, infection with a multidrug-resistant strain of *M. tuberculosis*, and the use of invasive ventilation in the course of COVID-19 treatment^(8,9)^.

One aspect that tuberculosis and COVID-19 have in common, in terms of their management, is the relevance of evaluation by imaging methods. Imaging (especially chest X-ray) is part of the initial investigation in suspected active tuberculosis and may support empirical treatment in patients with clinical or imaging findings suggestive of the diagnosis, even if mycobacteria have not been detected in sputum samples or by a specific rapid molecular test^(10)^. During the COVID-19 pandemic, imaging methods have been widely used, not only for diagnostic purposes but also to aid in the stratification of patients by severity. Although the use of imaging is not typically recommended as a screening tool, it is indicated in specific clinical contexts, such as in patients with a moderate-to-severe clinical presentation or who are at risk of progression, as well as where polymerase chain reaction is unavailable, to facilitate decisions regarding admission to the hospital or intensive care unit, to make differential diagnoses, and to evaluate comorbidities^(11-17)^. A number of systems of stratifying COVID-19 probability, based on the results of X-ray or computed tomography studies of the chest and with varying levels of interobserver agreement, have been disseminated over the course of the pandemic^(18)^.

Although there are still relevant arguments in the literature regarding whether the association between tuberculosis and COVID-19 is causal or coincidental, the fact is that the combination of the two is potentially deleterious, both from an individual and public health point of view. The situation in developing countries, where rates of *M. tuberculosis* infection are higher and resources are scarce, merits special attention^(3,19,20)^. In this context and in view of the relevant role that radiologists play in managing the two conditions, the article authored by Mançano et al.^(2)^ is of great interest.
